# Reoperation for Recurrence After Groin Hernia Repair in Adolescents: A Nationwide Register‐Based Cohort Study

**DOI:** 10.1002/wjs.12613

**Published:** 2025-05-07

**Authors:** Hugin Reistrup, Siv Fonnes, Andrea Joensen, Jacob Rosenberg

**Affiliations:** ^1^ Center for Perioperative Optimization Department of Surgery Copenhagen University Hospital—Herlev and Gentofte Herlev Denmark; ^2^ Section of Epidemiology Department of Public Health University of Copenhagen Copenhagen Denmark

**Keywords:** adolescents, femoral hernia, groin hernia, inguinal hernia, pediatric, recurrence

## Abstract

**Background:**

Although mesh‐based repairs are used in adults to reduce the risk of recurrence, their necessity in adolescents remains debated. Nonmesh repairs are often preferred in younger patients to avoid potential long‐term complications, but data on recurrence rates in this age group are limited. We aimed to assess the rate of reoperation for recurrence following primary groin hernia repair in adolescents aged 10–19 years.

**Methods:**

This was a register‐based cohort study covering three decades (1992–2022), utilizing data from the Danish National Patient Register linked to data from the Danish Civil Registration System, ensuring comprehensive nationwide coverage and complete follow‐up. The cumulative rate of reoperation for recurrence was estimated at 10 years of follow‐up. Cox proportional hazards regression analysis was used to compare the risk of reoperation for recurrence between mesh and nonmesh repairs. Outcomes included reoperation for recurrence, readmission, and mortality.

**Results:**

Among the 2404 included groins, most were male (80%), and the median age was 16 (IQR, 12–19) years. Most (99%) groin hernias were inguinal, and few (1%) were femoral. Of the inguinal repairs, 35% were mesh, 64% were nonmesh, and 1% were unspecified repairs. The follow‐up time was median 16 (IQR, 9–21) years. The cumulative rate of reoperation for recurrence across all inguinal repairs was 3.8% (95% CI, 3.0–4.9) after 10 years of follow‐up. For older adolescents aged 15–19 years, the cumulative rate of reoperation for recurrence after mesh and nonmesh repair was 2.7% (95% CI, 1.6–4.6) and 4.1% (95% CI, 2.6–6.7), respectively. Nonmesh repair had a higher adjusted hazard ratio of reoperation for recurrence compared with mesh repair (adjusted hazard ratio, 2.11; 95% CI, 1.05–4.23). For femoral repairs, most (67% [18/27]) were open nonmesh repairs, and few were reoperated for recurrence.

**Conclusion:**

The cumulative rate of reoperation for recurrence was low in adolescents. These findings suggest that nonmesh repair may be sufficient for primary groin hernia repair in adolescents, potentially avoiding the need for mesh implantation.

## Graphical abstract

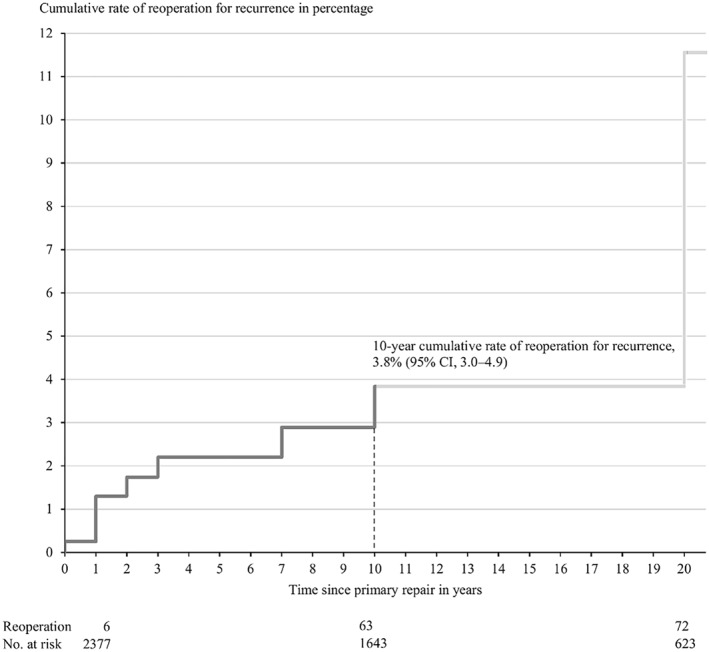

## 1 Introduction

Groin hernia is a common condition affecting people of all ages [1]. The literature on groin hernia is extensive. Comprehensive and regularly updated international guidelines exist for adults [2, 3], but little is known about the management of groin hernia in adolescents. For decades, recurrence has dominated the research field as a relevant endpoint [4, 5]. Mesh‐based repair has become a standard in adults as meshes have markedly lowered recurrence rates [6, 7], whereas nonmesh repair may be an option for young males with small indirect inguinal hernias [2, 8]. For young children, nonmesh repair is sufficient [9]. Still, there is a gap in data on recurrence as no large‐scale study with long‐term follow‐up has investigated reoperation for recurrence in adolescents [10]. Management of groin hernia in adolescents can challenge surgeons as adolescents are neither children nor adults [11]. Growth varies considerably among adolescents, making age an unreliable indicator of development, as growth stages differ between individuals [12, 13]. Traditionally, adults have been defined as patients aged 18 years and above in the general hernia literature [3], but a recent review has suggested expanding the upper age limit of adolescents with groin hernia to (and including) 19 years due to continued growth of the pelvis and inguinal ligaments after age 17 [14]. Implanting foreign bodies like synthetic meshes in young patients seems undesirable unless strictly indicated [14]. In young adults, mesh implantation may increase the risk of chronic pain [15], and although data on patient‐reported outcomes in adolescents are limited [10], surgeons managing adolescents prefer to avoid synthetic mesh implantation in this age group [11]. We hypothesized that nonmesh repair is sufficient with an acceptable risk of reoperation for recurrence after primary groin hernia repair in adolescents.

With the present study, we aimed to assess the rate of reoperation for recurrence after primary inguinal and femoral hernia repair in adolescents aged 10–19 years.

## 2 Methods

This was a nationwide register‐based cohort study of adolescents aged 10–19 years undergoing primary groin hernia repair in Denmark. The study was reported according to the REporting of studies Conducted using Observational Routinely‐collected health Data (RECORD) statement [16]. Data on repairs were obtained from the Danish National Patient Register (DNPR) from January 1, 1992, to December 31, 2022, including a lookback period from January 1, 1977, to December 31, 1991, to ensure that only primary repairs were included. Patients were followed until reoperation for recurrence, emigration, death, or the end of the study period (December 31, 2022), whichever came first. The DNPR is an administrative nationwide health register routinely registering all public hospital contacts, including surgical procedures, in Denmark since 1977, and including private hospitals and clinics since 2003 [17]. The study population was generated by the Danish Health Data Authority from data in the DNPR that also linked person‐level data with the Danish Civil Registration System (CRS) [18]. By linking the registers, complete follow‐up is possible as patients can be censored in case of emigration or death, enabling time‐dependent analyses. All variables on patient and hernia characteristics in this study came from the DNPR, whereas the variables on death and emigration came from the CRS. The investigators had access to pseudonymized data on all hospital contacts (public and private) and data from the CRS during the study period for patients fulfilling the inclusion criteria.

We included patients undergoing any primary groin hernia repair (inguinal and femoral) at the age of 10–19 years. We excluded groins with repairs performed before age 10 years and groins with primary repairs performed at age 20 years or older in the study period (1992–2022). To ensure that only primary repairs were identified, we also excluded groins with repairs performed at age 10 years or older in the lookback period (1977–1991). Repairs were identified in the DNPR via all inguinal and femoral hernia surgical procedure codes in the Danish Classification of Surgical Operations and Therapies (used in 1971–1995) [19] and the Nordic Medico‐Statistical Classification of Surgical Operations (NOMESCO) (used in 1996–present) (Supporting Information S1: Online Resource S1) [20]. The surgical procedure codes for inguinal and femoral hernia in the DNPR have previously been validated [21]. The surgical procedure codes can be accompanied by a supplemental code on laterality, describing the repair as right, left, or bilateral.

The study investigated outcomes after primary groin hernia repair at age 10–19 years. The primary outcome was the cumulative rate of reoperation for recurrence after inguinal hernia repair, which was defined as the second repair on the same side as the primary repair, registered with a surgical procedure code in the DNPR (Supporting Information S1: Online Resource S1). Reoperation was used as proxy for recurrence. The secondary outcomes were 30‐ and 90‐day all‐cause readmissions and 90‐day all‐cause mortality.

In this study, data were reported as individual groins and not patients. The supplemental code on laterality (right/left/bilateral) is not mandatory for hospitals to report to the DNPR. Consequently, data on laterality are sometimes missing and prohibit complete distinction of laterality for all registered repairs impeding accurate follow‐up on reoperation for recurrence. Therefore, we only included groins where laterality was certain for both the primary and, when applicable, for the secondary repair for a given groin. This ensured that all reoperations were on the same side as the primary repair. To assess the risk of confounding, differences in the registration rate of laterality were investigated by comparing hospitals and year of registration, respectively, and no difference was found (data not shown). To address selection bias, a cohort including all patients undergoing inguinal hernia repair regardless of the certainty of laterality is presented in Supporting Information S1: Online Resource S2.

**FIGURE 1:**
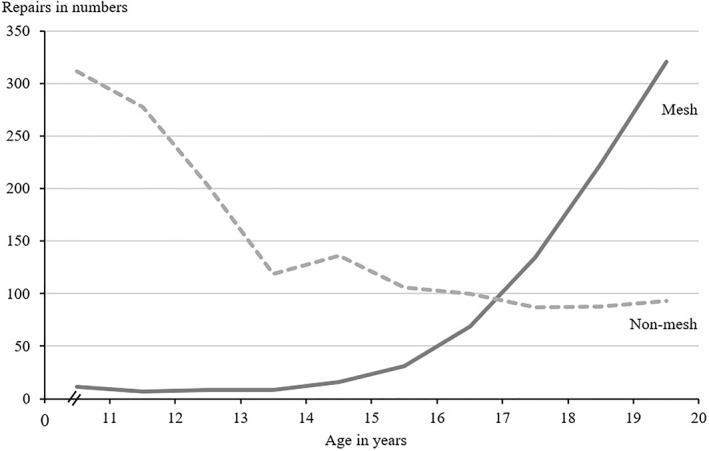
Number of primary inguinal hernia repairs with registered, certain laterality in adolescents in Denmark from 1992 to 2022 (*y*‐axis) and age in years at repair (*x*‐axis). Data stratified by an operative approach. Nonmesh repairs are depicted with a dashed line.

Statistical analyses were conducted in SAS, version 9.4, SAS Institute Inc., Cary, NC, USA. The study size was determined by the number of groins meeting the inclusion criteria during the study period. Histograms and QQ plots were used to evaluate the distribution of continuous data. Nonnormally distributed continuous data were presented as median and interquartile range. Crude reoperation rates were presented with 95% confidence intervals (CI) and calculated as the number of secondary repairs divided by the number of primary repairs. The cumulative rate of reoperation for recurrence was calculated as the number of secondary repairs divided by the number at risk at selected time points. Subgroup analyses were conducted on young (10–14 years) and old (15–19 years) adolescent age groups, respectively. Cox proportional hazards regression analysis was conducted to assess the hazard of reoperation for recurrence after mesh compared with nonmesh repair in a pooled age group of older adolescents aged 15–19 years, as mesh‐based repairs were rare before age 15 (Figure 1). The estimate was presented as a hazard ratio (HR) with corresponding 95% CI. Nonmesh repairs were used as the reference group. The analysis was adjusted for sex as the risk of reoperation for recurrence differs between females and males [22]. If data points contained data on fewer than five observations, the exact values were not reported to maintain patient anonymity in accordance with regulations by the Danish Health Data Authority [23].

This study was conducted according to the principles in the Declaration of Helsinki [24]. The study was approved by the Danish Data Protection Agency (P‐2023‐284). Data were supplied by the Danish Health Data Authority (FSEID‐00006620). Approval by the Ethics Committees for the Capital Region of Denmark was not required [25].

## 3 Results

### 3.1 Selection

**FIGURE 2:**
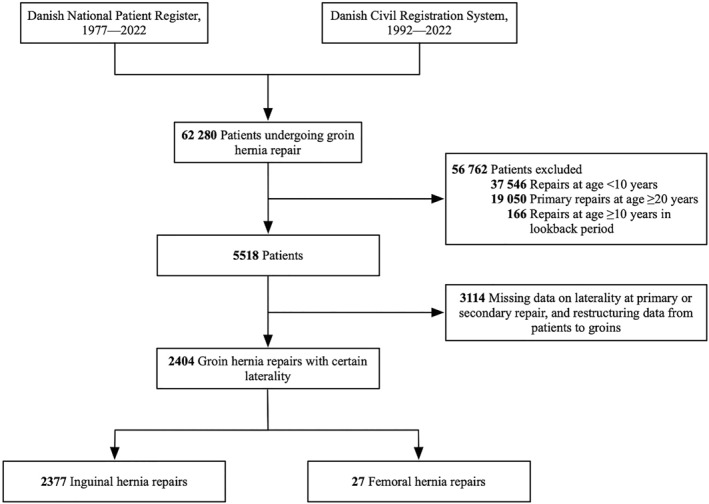
Flowchart of inclusion and exclusion. The study period was 1992–2022, and the lookback period was 1977–1991. The 62,280 patients undergoing groin hernia repair represent the raw dataset obtained from the Danish Health Data Authority before the data cleansing process.

During a 30‐year period from 1992 to 2022, a total of 5518 patients underwent a groin hernia repair at age 10–19 years and were eligible for inclusion (Figure 2). Laterality of the repairs was certain in 2404 groins in 2363 patients, and these were included for analysis. There was no indication of selection bias due to missing data on the patient characteristics; however, there were some differences in the surgical characteristics (Supporting Information S1: Online Resource S2). Close to all (99% [2377 of 2404]) primary repairs were for inguinal hernias and few (1% [27 of 2404]) for femoral hernias. As all data were obtained from the DNPR and CRS, 100% follow‐up was achieved.

### 3.2 Inguinal Hernia Repairs

#### 3.2.1 Characteristics

In total, 2377 groins underwent a primary inguinal hernia repair and their characteristics are presented in Table 1. Most were males (81% [1922 of 2377]) and the median age was 16 (IQR, 12–19) years at primary repair. There were 35% (829 of 2377) mesh repairs, 64% (1522 of 2377) nonmesh repairs, and 1% (26 of 2377) unspecified mesh/nonmesh repairs. In total, 82% (1938 of 2377) of repairs were performed as outpatient, same‐day surgery, whereas 14% (328 of 2377) were admitted for one night only. During the study period, the number of mesh repairs was low before age 15 but increased markedly thereafter, whereas the number of nonmesh repairs decreased steadily, reaching a plateau at approximately age 17 (Figure 1). The number of mesh and nonmesh repairs intersected at age 17. Follow‐up time was median 16 (IQR, 9–21) years.

**TABLE 1:** Characteristics of adolescents (10–19 years) undergoing primary inguinal hernia repair.^a^

Characteristics	
Patient characteristics	
Groins, no.	2377
Sex	
Male	1922 (81)
Female	455 (19)
Age, median (IQR), years	16 (12–19)
Hernia laterality	
Unilateral	2365 (99.5)
Right	1435 (61)
Left	930 (39)
Bilateral	12 (0.5)
Surgical characteristics	
Operative approach	
Mesh	829 (35)
Open	578 (70)
Laparoscopic	251 (30)
Nonmesh	1522 (64)
Open	1522 (100)
Unspecified mesh/nonmesh	26 (1)

^a^ Data are given as the number (percentage) of groins, unless otherwise indicated.

#### 3.2.2 Reoperation for Recurrence

The crude rate of reoperation for recurrence after primary inguinal hernia repair for mesh and nonmesh repair was 1.9% [95% CI, 1.1–3.1] and 3.6% [95% CI, 2.7–4.7], respectively. The crude rates of reoperation for recurrence for age subgroups are shown in Table 2.

**TABLE 2:** Crude reoperation rates for primary inguinal hernia repair in adolescents.

Age group	All repairs	Mesh	Nonmesh	Unspecified mesh/nonmesh
10–19 years				
Groins, no. (%)	2377 (100)	829 (35)	1522 (64)	26 (1)
Reoperation rate, no. (% [95% CI])	NR	16 (1.9 [1.1–3.1])	55 (3.6 [2.7–4.7])	NR
Follow‐up time, median (IQR), years	16 (9–21)	13 (8–19)	17 (10–21)	NR
10–14 years				
Groins, no. (%)	1110 (100)	50 (5)	1048 (94)	12 (1)
Reoperation rate, no. (% [95% CI])	NR	NR	36 (3.4 [2.4–4.7])	NR
Follow‐up time, median (IQR), years	15 (9–21)	15 (7–19)	15 (9–21)	NR
15–19 years				
Groins, no. (%)	1267 (100)	779 (61)	474 (37)	14 (1)
Reoperation rate, no. (% [95% CI])	NR	NR	19 (4.0 [2.4–6.2])	NR
Follow‐up time, median (IQR), years	16 (9–21)	13 (8–19)	19 (13–22)	NR

*Note:* NR, not reported in accordance with regulations by the Danish Health Data Authority as there were fewer than five observations or where disclosing information would enable the calculation of data points with fewer than five observations [23].

**FIGURE 3:**
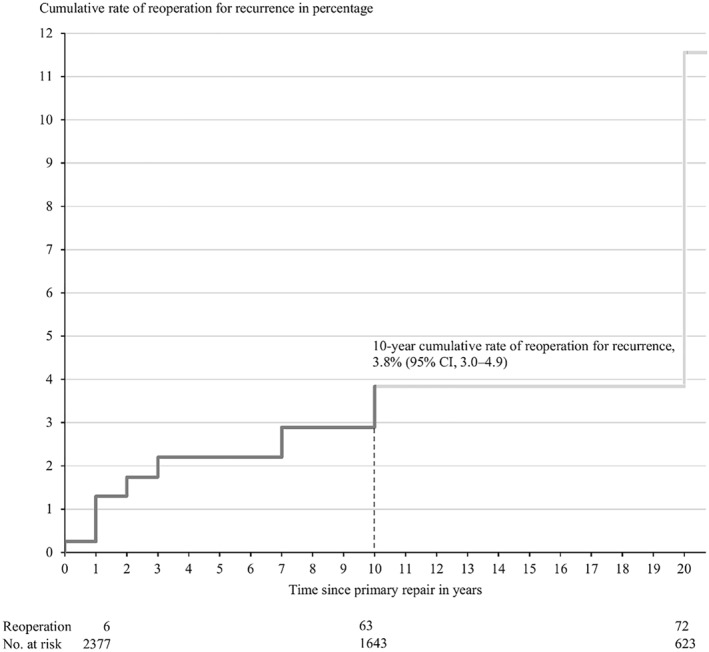
Cumulative rate of reoperation for recurrence in percentage after primary inguinal hernia repair in adolescents using any surgical procedure code (*y*‐axis). The plot is translucent from 10 to 20 years due to the low number at risk in this follow‐up period. The plot only depicts changes in the cumulative rate of reoperation where there are five or more events (recurrences) per regulations by the Danish Health Data Authority [23].

The cumulative rate of reoperation for recurrence across all repairs was 3.8% (95% CI, 3.0–4.9) after 10 years of follow‐up (Figure 3). Subgroup analyses on older adolescents aged 15–19 years after 10 years of follow‐up showed a cumulative rate of reoperation for recurrence for mesh and nonmesh repair of 2.7% (95% CI, 1.6–4.6) and 4.1% (95% CI, 2.6–6.7), respectively. When pooling older adolescents aged 15–19 years in unadjusted Cox proportional hazards regression analysis, nonmesh repair showed a higher hazard ratio of reoperation for recurrence compared with mesh repair (unadjusted HR, 2.05 [95% CI, 1.03–4.09]). When adjusting for sex, the hazard ratio increased marginally (adjusted HR, 2.11 [95% CI, 1.05–4.23]).

#### 3.2.3 Readmission and Mortality

The 30‐ and 90‐day all‐cause readmission rates across all repairs were 1.3% (32 of 2377) and 3.4% (82 of 2377), respectively. The 90‐day all‐cause mortality rate across all repairs was 0% (0 of 2377).

### 3.3 Femoral Hernia Repairs

A total of 27 groins underwent a primary femoral hernia repair; 67% (18 of 27) were female, and 33% (9 of 27) were male. Among the 27 operated groins, 67% (18 of 27) had an open nonmesh repair. All repairs were unilateral. There were less than five reoperations for recurrence in the study period. More detailed data could not be reported due to regulations on the reporting of small sample sizes to uphold patient anonymity [23].

## 4 Discussion

This study found a low cumulative rate of reoperation for recurrence across all repairs after primary inguinal hernia repair in adolescents aged 10–19 years. For older adolescents aged 15–19 years, nonmesh repair had a higher hazard ratio of reoperation for recurrence compared with mesh repair, though the estimate was uncertain. Femoral hernias were rare in adolescents, and reoperations for recurrence were few. These findings support that the nonmesh repair of inguinal hernia in adolescents seems sufficient, thereby avoiding the implantation of a foreign body in young patients. Also, nonmesh repair of femoral hernia in adolescents may be sufficient.

We found a cumulative rate of reoperation for recurrence after primary inguinal hernia repair of 3.8% after 10 years of follow‐up. The findings in this study support the limited existing literature on the subject while expanding the body of evidence on adolescents substantially. A recent meta‐analysis including 21 studies on 3953 groin hernia repairs in adolescents aged 10–17 years found low incidences of recurrence of approximately 2% across all surgical techniques [10]. Most included studies were retrospective, most investigated open nonmesh repair, and follow‐up times varied considerably and were reported insufficiently. Only seven studies investigated adolescents exclusively [26, 27, 28, 29, 30, 31, 32], underlining the lack of data in the literature prior to the current study. In subgroup analysis on 15‐ to 19‐year‐old adolescents, we found a higher hazard ratio of reoperation for recurrence after nonmesh compared with mesh repair (adjusted HR, 2.11 [95% CI, 1.05–4.23]). This subgroup analysis was conducted because pooling patients aged 10–19 years may be misleading as the anatomy of a 10‐year‐old may differ substantially from that of a 19‐year‐old [33]. More interesting is perhaps the recurrence rate after nonmesh repair in older adolescents. Our study found a low and acceptable cumulative rate of reoperation for recurrence after 10 years of follow‐up among older adolescents undergoing nonmesh repair (4.1% [95% CI, 2.6–6.7]). This implies that approximately 96% of older adolescents remain recurrence‐free 10 years after primary repair. This is in line with previous studies on young adults [8, 34], suggesting that nonmesh repair may also be feasible in selected young adults with inguinal hernia. A recent large register‐based study found that the rate of reoperation for recurrence after inguinal repair in 16 to 17‐year‐olds was 2.5% (95% CI, 1.6–2.4) after 5 years, with no information on the repair methods used [35].

This study found that few adolescents were readmitted to hospital and that none died within 90 days. This is in line with a register‐based study also using data from the DNPR on 2476 children undergoing inguinal hernia repair [36]. They found a 30‐day readmission rate of 2.1% and no hernia‐related deaths. This finding suggests that inguinal hernia repair in adolescents appears to be safe.

This study has several strengths. To our knowledge, this is the first‐of‐its‐kind nationwide study on groin hernia repair in adolescents with complete follow‐up over a 30‐year period. We censored for death and emigration in time‐dependent analyses, increasing the accuracy of the estimates. It has a high external validity regarding adolescents with groin hernia due to the nationwide design and broad inclusion criteria (primary groin hernia repair in adolescents aged 10–19 years). The results are therefore applicable to adolescent patients with groin hernia managed in comparable settings. This study also has limitations. Reoperation was used as a proxy for recurrence, though it might underestimate the true recurrence rate [37]. Distinguishing between medial and lateral inguinal hernia is not possible in the DNPR [21], though this is relevant as medial hernias are known to recur more often than lateral hernias [38]. Laterality (right/left/bilateral) was missing for more than half of the identified patients eligible for inclusion as it is not mandatory to register in the DNPR. Information on laterality is necessary to determine if a secondary repair is a reoperation on the same side or a primary repair on the contralateral side. To address this, we only analyzed data on groin repairs with certain laterality; hence, the rate of reoperation for recurrence was perhaps underestimated, though the distribution of the registration of laterality did not seem biased by the geographical location of hospitals, year of registration, or patient characteristics. This study was also limited by the data being routinely collected for the DNPR, preventing analyses on important operative characteristics that may affect recurrence rates like defect size, mesh type, mesh fixation, and patient‐reported outcomes such as pain [39]. Operative data, including laterality and detailed information on repair techniques (e.g., high ligation or primary tissue repair), are available in surgeon‐reported databases like the Danish Hernia Database [40], the Swedish Hernia Register [41], and Herniamed [42]. The Danish Hernia Database and the Swedish Hernia Register are national registries with high coverage rates of 93% [43] and 94% [44], respectively. However, as the Danish Hernia Database does not register data on patients under the age of 18 years, these data are unfortunately not available for adolescents. As groin hernias in adolescents are relatively rare, methods for assessing postoperative outcomes for larger cohorts of adolescents are limited. Therefore, we recommend that patients of all ages including adolescents should be registered in surgeon‐reported databases for long‐term follow‐up.

Nonmesh repair may be sufficient for primary groin hernia repair in adolescents which could have clinical implications as the evidence on mesh‐related complications is conflicting. For young patients, postoperative chronic pain may be the most worrisome complication after hernia surgery—a concern also raised by surgeons in relation to mesh‐based repairs [11]. For patients experiencing postoperative chronic pain, management can be difficult, escalating from an initial wait‐and‐see strategy to systemic painkillers, injections, and ultimately surgical treatment [45]. The cause of postoperative chronic pain is probably multifactorial, and the pathogenesis may vary between individuals, but the implantation of synthetic meshes in human tissue is a concern. Data have indicated that open mesh repair for inguinal hernia may cause postoperative chronic pain in young patients [15, 46], and the recently updated international guideline on groin hernia management suggests that simple annulorrhaphy could be sufficient in young men with small indirect inguinal hernia [2]. Still, evidence on chronic pain after inguinal hernia repair in the general adult population has not found a difference between mesh and nonmesh repair [47]. In theory, implanting a foreign material may be troublesome in growing tissue. The long‐term tension on the mesh and surrounding tissue occurring as the groin and pelvis grow during adolescence [33], and as the mesh shrinks with time [48], may contribute to chronic pain. Additionally, although no impact on fertility has been shown in adults [49], concerns remain regarding potential effects in young male patients after mesh implantation, which, to our knowledge, has not been studied in adolescents. To improve our understanding of potential long‐term harms after groin hernia repair in adolescents, future studies focusing on patient‐reported outcomes, including postoperative chronic pain [50], in young patients are encouraged.

## 5 Conclusion

The cumulative rate of reoperation for recurrence was low across all repairs after primary inguinal hernia repair in adolescents. Also, few femoral hernias were reoperated for recurrence, though the sample was small. Given the low rate of reoperation for recurrence and the potential complications associated with mesh implantation in this age group—where patients are still growing—these results suggest that nonmesh repair may be sufficient for repairing primary groin hernia in adolescents, avoiding foreign body implantation in 10‐ to 19‐year‐old patients.

## Author Contributions

**Hugin Reistrup:** conceptualization, data curation, formal analysis, funding acquisition, investigation, methodology, project administration, software, validation, visualization, writing – original draft. **Siv Fonnes:** conceptualization, data curation, formal analysis, investigation, methodology, software, supervision, validation, visualization, writing – review and editing. **Andrea Joensen:** conceptualization, data curation, formal analysis, investigation, methodology, software, validation, writing – review and editing. **Jacob Rosenberg:** conceptualization, investigation, methodology, supervision, writing – review and editing.

## Conflicts of Interest

The authors declare no conflicts of interest.

## Supporting information

Supporting Information S1

## Data Availability

The authors have nothing to report.
