# Supplementary material for: Reoperation for Recurrence After Groin Hernia Repair in Adolescents: A Nationwide Register‐Based Cohort Study
Source: World J Surg. 2025 May 7;49(6):1441–8. doi: 10.1002/wjs.12613 (PMC12134194; doi:10.1002/wjs.12613)
Supplement: Supplementary file 1 — Supporting Information S1 [file WJS-49-1441-s001.docx]

# Electronic Supplementary Material

**Article title:**
Reoperation for recurrence after groin hernia repair in adolescents: a nationwide register-based cohort study

**Journal name:**
World Journal of Surgery

**Author names:**
Hugin Reistrup^1^, Siv Fonnes^1^, Andrea Joensen^2^, Jacob Rosenberg^1^

**Affiliation:**^1^ Center for Perioperative Optimization, Department of Surgery, Copenhagen University Hospital - Herlev and Gentofte, Borgmester Ib Juuls Vej 1, DK-2730 Herlev, Denmark

^2^ Section of Epidemiology, Department of Public Health, University of Copenhagen, Øster Farimagsgade 5, bd. 24, DK-1014 Copenhagen, Denmark

**Corresponding author:**Hugin Reistrup
E-mail: hugin.reistrup@gmail.com

**Online Resource 1. Surgical procedure codes for groin hernia repairs**

^a^ Non-mesh repair, as mesh was seldom used in laparoscopy in Denmark prior to 1995.

| **Hernia type** | | **Code** | **Description** | **Mesh (+),**  **non-mesh (-),**  **unspecified (?)** | **Open (O), laparoscopic (L)** |
| --- | --- | --- | --- | --- | --- |
| **The Danish Classification of Surgical Operations and Therapies (1971–1995)** | | | | | |
|  | Inguinal | 40620 | Inguinal hernia operation | - | O |
|  |  | 40621 | Endoscopic inguinal hernia operation | -^a^ | L |
|  |  | 40640 | Preperitoneal inguinal hernia operation | + | O |
|  |  | 42000 | Inguinal hernia operation | - | O |
|  | Femoral | 40660 | Femoral hernia operation | - | O |
|  |  | 42100 | Femoral hernia operation | - | O |
|  | Unspecified | 40740 | Groin hernia operation | - | O |
|  |  | 42900 | Groin hernia operation | - | O |
|  |  | 40760 | Groin hernia operation with fascia repair | - | O |
|  |  | 40800 | Groin hernia operation with synthetic material | + | O |
|  |  | 40801 | Laparoscopic groin hernia operation with synthetic material | + | L |
|  |  | 40840 | Groin hernia operation with synthetic material | + | O |
|  |  | 42810 | Groin hernia operation with biologic material | + | O |
| **The Nordic Medico-Statistical Classification of Surgical Operations (NOMESCO) (1996–present)** | | | | | |
|  | Inguinal | KJAB00 | Inguinal hernia operation | - | O |
|  |  | KJAB10 | Operation for inguinal hernia with plasty | - | O |
|  |  | KJAB11 | Laparoscopic inguinal hernia operation | + | L |
|  |  | KJAB20 | Inguinal hernia operation with fascia repair | - | O |
|  |  | KJAB30 | Inguinal hernia operation with synthetic material | + | O |
|  |  | KJAB40 | Inguinal hernia operation with abdominal wall plasty through laparotomy | - | O |
|  |  | KJAB96 | Other inguinal hernia operation | ? | O |
|  |  | KJAB97 | Other laparoscopic inguinal hernia operation | + | L |
|  | Femoral | KJAC10 | Femoral hernia operation | - | O |
|  |  | KJAC11 | Laparoscopic femoral hernia operation | + | L |
|  |  | KJAC30 | Femoral hernia operation with synthetic material | + | O |
|  |  | KJAC40 | Femoral hernia operation with abdominal wall plasty through laparotomy | - | O |
|  |  | KJAC96 | Other femoral hernia operation | ? | O |
|  |  | KJAC97 | Other laparoscopic femoral hernia operation | + | L |

**Online Resource 2. Characteristics for primary inguinal hernia repairs for the cohort with certain laterality and the total cohort with both certain and uncertain laterality^a^**

Abbreviations: NA, not available due to missing information on laterality for a part of the cohort

^a^ Data are given as number (percentage) of groins for the cohort with certain laterality and as number (percentage) of patients for the total cohort with both certain and uncertain laterality, unless otherwise indicated; ^b^ Cohort including groins with both certain and uncertain laterality

| **Characteristics** | | | | | **Certain laterality** | **Total^b^** |
| --- | --- | --- | --- | --- | --- | --- |
|  |  |  |  |  | **(groins)** | **(patients)** |
| **Patient characteristics** | | | | |  |  |
|  | Patients, No. | | | | 2363 | 5377 |
|  | Groins, No. | | | | 2377 | NA |
|  | Sex | | | |  |  |
|  |  | Male | | | 1922 (81) | 4432 (82) |
|  |  | Female | | | 455 (19) | 945 (18) |
|  | Age, median (IQR), y | | | | 16 (12–19) | 15 (12–18) |
| **Surgical characteristics** | | | | |  |  |
|  | Operative approach | | | |  |  |
|  |  | | Mesh | | 829 (35) | 1398 (26) |
|  |  | |  | Open | 578 (70) | 984 (70) |
|  |  | |  | Laparoscopic | 251 (30) | 414 (30) |
|  |  | | Non-mesh | | 1522 (64) | 3917 (73) |
|  |  | |  | Open | 1522 (100) | 3910 (99.8) |
|  |  | |  | Laparoscopic | 0 (0) | 7 (0.2) |
|  |  | | Unspecified mesh/non-mesh | | 26 (1) | 62 (1) |
|  | Follow-up time, median (IQR), y | | | | 16 (9–21) | 19 (12–25) |
